# Reinforcement learning-trained optimisers and Bayesian optimisation for online particle accelerator tuning

**DOI:** 10.1038/s41598-024-66263-y

**Published:** 2024-07-08

**Authors:** Jan Kaiser, Chenran Xu, Annika Eichler, Andrea Santamaria Garcia, Oliver Stein, Erik Bründermann, Willi Kuropka, Hannes Dinter, Frank Mayet, Thomas Vinatier, Florian Burkart, Holger Schlarb

**Affiliations:** 1 Deutsches Elektronen-Synchrotron DESY, Hamburg, Germany; 2 Karlsruhe Institute of Technology KIT, Karlsruhe, Germany; 3 Hamburg University of Technology, 21073 Hamburg, Germany

**Keywords:** Experimental particle physics, Computational science, Computer science, Information technology

## Abstract

Online tuning of particle accelerators is a complex optimisation problem that continues to require manual intervention by experienced human operators. Autonomous tuning is a rapidly expanding field of research, where learning-based methods like Bayesian optimisation (BO) hold great promise in improving plant performance and reducing tuning times. At the same time, reinforcement learning (RL) is a capable method of learning intelligent controllers, and recent work shows that RL can also be used to train domain-specialised optimisers in so-called reinforcement learning-trained optimisation (RLO). In parallel efforts, both algorithms have found successful adoption in particle accelerator tuning. Here we present a comparative case study, assessing the performance of both algorithms while providing a nuanced analysis of the merits and the practical challenges involved in deploying them to real-world facilities. Our results will help practitioners choose a suitable learning-based tuning algorithm for their tuning tasks, accelerating the adoption of autonomous tuning algorithms, ultimately improving the availability of particle accelerators and pushing their operational limits.

## Introduction

Particle accelerators are instrumental in facilitating scientific and technological progress, from fundamental physics research and the study of proteins to cancer therapy and numerous other domains. For their successful operation, it is critical that these facilities achieve predefined performance metrics. These are reached through online tuning, i.e. the optimisation of the plant and its subsystems towards a desired system state. Tuning these systems is a challenging optimisation problem due to the non-linear and often dynamic correlations among numerous tuning parameters. Moreover, the inherent noise in real-world measurements, the time-consuming data acquisition, and the high costs associated with system downtime make the tuning of particle accelerators particularly challenging.

To date, online tuning continues to be performed mostly manually, relying on the experience of expert human operators. This leads to suboptimal solutions that are time-intensive to attain and difficult to reproduce.

To reduce downtime and push the limits of their operational capabilities, efforts are made to develop autonomous accelerator tuning solutions^1^. Existing approaches can improve optimisation results, reproducibility, and reliability for some tuning tasks, but come with their own drawbacks. For example, while grid search and random search are reliable and highly reproducible approaches, they require many samples. As a result, these methods become impractical for online tuning, where the cost per sample is often prohibitively high. Other approaches from the field of numerical optimisation can reduce the number of required samples and have been successfully applied to tuning tasks^2,3^. While these approaches show promising results, due to the so-called *curse of dimensionality*^4^, their performance drops as the number of tuning dimensions increases and objective functions become more complex. Furthermore, many of these methods are sensitive to noise^3^, which is omnipresent in real-world measurements.

Learning-based methods have emerged as promising solutions capable of sample-efficient, high-dimensional optimisation under real-world conditions. As one of the most successful learning-based optimisation algorithms, Bayesian optimisation (BO)^5^ has already found widespread adoption in the accelerator community with promising results^6–11^. With BO, the number of samples required for a successful optimisation is reduced by learning a probabilistic surrogate model of the objective function at the time of optimisation and using this surrogate model to determine where to sample the objective function next.

In the field of control, reinforcement learning (RL) is increasingly used to train intelligent controllers for various application domains, including particle accelerators^12,13^. In more recent research, the fact that continued optimisation of a dynamic function can be considered to be equivalent to control is exploited by using RL to train domain-specialised optimisation algorithms. This so-called *reinforcement learning-trained optimisation (RLO)*^14–16^ falls into a larger research field known as *optimiser learning*^14–17^. The paradigm of RLO can be applied to training optimisers specialised in accelerator tuning tasks^18–20^.

Adjacent to these primarily learning-based algorithms, there is a line of research, where data-driven models are combined with classical control algorithms for particle accelerator tuning and control. In this line of work, Proportional-Integral (PI) controllers have been coupled with neural network (NN) predictors to achieve control over a wider range of conditions^21^, NN models have been integrated as the system model with model predictive control (MPC)^22^, and the performance of extremum seeking (ES) was improved on high-dimensional control tasks through the use of an NN predictor providing an initial guess close to the real optimum from where ES can efficiently perform the rest of the tuning^23^. Unlike with RLO and BO, the focus of these hybrid approaches mostly lies in learning an expressive and accurate machine learning (ML) model, rather than the applied optimisation algorithm.

As such, RLO and BO are two state-of-the-art learning-based methods for developing optimisation algorithms for particle accelerator tuning. However, in practice, RL is mostly considered for continuous control problems, while BO is more commonly considered for tuning. We believe that under the paradigm of RLO, RL presents a highly under-considered alternative to BO, which is better suited to certain tuning tasks, and provides value beyond the static tuning case in dynamic tuning. To date, there is no prior work providing data-driven comparisons of these two algorithms and their practical merits and challenges when applying them to real-world facilities such as particle accelerators.

In this work, we study RLO and BO for tuning a subsystem of a particle accelerator and compare them in terms of the achieved optimisation result, their convergence speed, and the practical challenges one is likely to encounter when deploying these methods to accelerator tuning tasks. Specifically, we compare two common setups of these algorithms, where the policy model of RLO is trained in simulation because training directly on the accelerator is not feasible as a result of limited beamtime and large number of required training samples, while BO is evaluated without prior information, learning the Gaussian process GP model entirely online. Although these are the most common setups with which RLO and BO are applied to online tuning in their basic form, task-specific modifications are possible and discussed in “Discussion”. To ensure the reliability of our results, we combine a significant number of simulations with real-world measurements. Based on our findings, we ascertain the practical merits and challenges of each tuning method with the aim of guiding the choice of algorithm for online tuning of particle accelerators. Additionally, RLO and BO have gained increased attention with other complex real-world plants, such as nuclear fusion reactors, optical radio telescopes, and grid-interactive buildings^24–38^. These face similar challenges when deploying autonomous tuning algorithms, which is why we believe that this study can also provide insights beyond the field of particle accelerators. Considering RLO as a powerful alternative to BO for tuning tasks and benchmarking both methods for the same problem in simulation and in the real world, this work provides a useful study case that can help accelerate the deployment of these methods to day-to-day operations.

## Results

**Figure 1:**
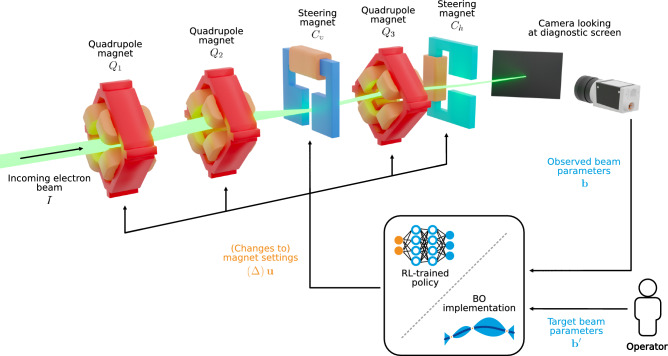
Simplified 3D illustration of the considered transverse beam tuning task at the ARES particle accelerator. The accelerator section consists of three quadrupole magnets and two steering magnets, followed by a diagnostic screen. The measured beam $\varvec{b}$ and the desired beam $\varvec{b}'$ are provided to the algorithm performing the tuning. In the case of BO, they are used to compute the objective. In the case of RL, they are provided along with the magnet settings as input to the policy and are used to calculate the reward. Both algorithms output either the next settings to the magnets $\varvec{u}$ or a change to the magnets $\Delta \varvec{u}$.

In this study, we consider as a benchmark a recurring beam tuning task which is ubiquitous across linear particle accelerators and frequently performed during start-up and operation mode changes, where the goal is to focus and steer the electron beam on a diagnostic screen. While this task can be very time-consuming in manual tuning, it is also well-defined, making it suitable as a proof-of-concept application for RLO and BO. For the study, we use the specific magnet lattice of a section of the ARES (Accelerator Research Experiment at SINBAD) particle accelerator^39,40^ at DESY in Hamburg, Germany, one of the leading accelerator centres worldwide. From here on, we refer to this section as the *accelerator section*. An illustration of the accelerator section is shown in Fig. 1. Further details on ARES and the accelerator section are given in “ARES particle accelerator section”. The lattice of the accelerator section is in downstream order composed of two quadrupole focusing magnets $Q_1$ and $Q_2$, a vertical steering magnet $C_v$, a third quadrupole focusing magnet $Q_3$, and a horizontal steering magnet $C_h$. Downstream of the magnets, there is a diagnostic screen capturing a transverse image of the electron beam. A Gaussian distribution $\varvec{b}= \left( \mu _x, \sigma _x, \mu _y, \sigma _y \right)$ is fitted to the observed image, where $\mu _{x, y}$ denote the transverse beam positions and $\sigma _{x,y}$ denote the transverse beam sizes. The goal of the tuning task is to adjust the quadrupole magnets’ field strengths *k* and steering magnets’ steering angles $\alpha$ to achieve a target beam $\varvec{b}'$ chosen by a human operator. We denote the *actuators*, here the magnet settings to be changed by the algorithm, as $\varvec{u}= \left( k_{Q_1}, k_{Q_2}, \alpha _{C_v}, k_{Q_3}, \alpha _{C_h} \right)$. The optimisation problem can be formalised as minimising the objective

1 $$\begin{aligned} \min _{\varvec{u}} O \left( \varvec{u}\mid M, I\right) = \min D \left( \varvec{b}\left( \varvec{u}\mid M, I\right) , \varvec{b}'\right) , \end{aligned}$$

which for the benchmark tuning task is defined as the difference *D* between the target beam $\varvec{b}'$ and the observed beam $\varvec{b}$. The observed beam $\varvec{b}$ is determined by the beam dynamics, which depend on the actuators $\varvec{u}$, and environmental factors, such as the magnet misalignments $M$ and the incoming beam $I$ to the accelerator section. Together with the target beam $\varvec{b}'$, these define the *state* of the environment. With most real-world tuning tasks, the state is only *partially observable*. In the case of the benchmark task, the magnet misalignments and the incoming beam cannot be easily measured or controlled, and are therefore part of the environment’s hidden state. As a measure of difference between the observed beam $\varvec{b}$ and the target beam $\varvec{b}'$, we use the mean absolute error (MAE) defined as

2 $$\begin{aligned} D_{\text{MAE}} (\varvec{b}, \varvec{b}^{\prime}) = \frac{1}{4} \sum _{i=1}^{4} \left| \varvec{b}^{(i)} - \varvec{b}^{\prime(i)} \right| , \end{aligned}$$

i.e. the mean of the absolute value of the beam parameter differences over all four beam parameters, where $\varvec{b}^{(i)}$ denotes the *i*-th element of $\varvec{b}$.

In our analysis, an RLO policy trained for previous work^19^ was used. It is described in “Reinforcement learning”. Further, a standard implementation of BO with a Gaussian process (GP) model^41^, matching the state of the art of BO implementations for particle accelerator tuning was used. It is outlined in “Bayesian optimisation”. In addition to the studied RLO and BO solutions, we consider random search, Nelder–Mead Simplex optimisation^42^, and ES^43^ as baselines for randomised and heuristic optimisation algorithms. They are presented in “Random search”, “Nelder–Mead simplex”, and “Extremum seeking”.

### Simulation study

**Figure 2:**
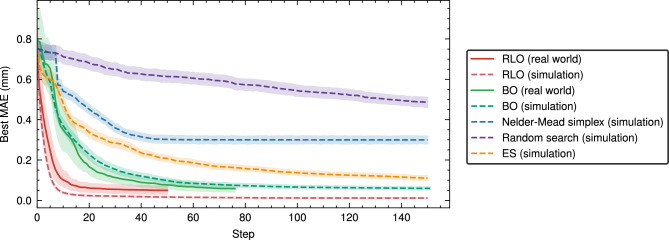
Beam difference convergence plots for different optimisation algorithms. The MAEs of the beam parameters to the target beam are shown for real-world experiments (solid lines) and simulated experiments (dashed lines), averaged over all trials. The envelopes show the 95% confidence intervals of the beam differences averaged over all trials. Specifically, the best beam differences encountered up to each step are shown, i.e. the beam differences that one would return to if the optimisation was terminated in the respective step. We refer to this metric as the *Best MAE*.

For the simulation study, we consider a fixed set of 300 randomly generated environment states, each defined by a target beam $\varvec{b}'$, an incoming beam $I$ entering the accelerator section from upstream, and transverse misalignments of the quadrupole magnets and the diagnostic screen $M$. We refer to these instances of the environment state as *trials*, defined in Eq. (3). The results of the simulation study over RLO and BO, as well as the baseline algorithms, random search, Nelder–Mead Simplex and ES, are summarised in Table 1. Box plots further illustrating these results can be found in Supplementary Fig. S1 to S3. Convergence plots for all algorithms are shown in Fig. 2.

We find that the learning-based algorithms RLO and BO outperform all baselines in terms of the optimisation result, achieving a final beam difference *D* at least 6 times smaller than that achieved by Nelder–Mead simplex and half of that achieved by ES. Furthermore, RLO achieves a median final beam difference *D* of 4$\upmu$m, which is more than an order of magnitude smaller than the one achieved by BO. The final beam difference achieved by RLO is smaller than the one achieved by BO in 96% of the trials. Note that the final beam difference achieved by RLO is also smaller than the real-world diagnostic screen’s measurement accuracy of 20$\upmu$m.

Given the measurement accuracy, we define a threshold $\epsilon =$ 40 $\upmu$m as twice the measurement accuracy and construct two metrics to measure the optimisation speed. We define *steps to target* as the number of steps until the observed beam parameters differ less than an average of $\epsilon$ from the target beam parameters, and *steps to convergence* as the number of steps after which the average of the beam parameters never changes by more than $\epsilon$. We observe that RLO always converges and manages to do so close to the target in 92% of trials. BO also converges on all trials, but only does so close to the target in 43% of trials, taking about 3–4 times longer to do so. The reason for this is that BO explores the optimisation space instead of fully converging toward the target beam. This effect can also be seen in Supplementary Fig. S6 and S7, where BO can be seen to explore a larger region of the objective space while finding a similar optimum to RLO. It is possible to suppress this behaviour by using an acquisition function that favours exploitation, but our experiments have shown that such acquisition functions are more susceptible to objective functions with multiple prominent local optima.

**Figure 3:**
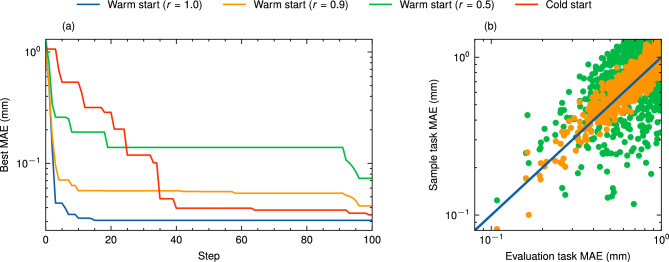
Bayesian optimisation results with historic samples. (**a**) The progress of BO evaluation runs using 100 samples with different correlation coefficients *r* to warm-start the GP model, compared to a cold start optimisation. The correlation coefficient is a metric of the similarity between the old and new task, where the incoming beam was changed. When the samples correspond to the same incoming beam, $r = {1}$. (**b**) Scatter plot visualising the objective value of the same randomised parameter settings in the old and new tasks. The MAEs are shown in log-scale for better visualisation.

It needs to be pointed out that the convergence speed difference is mostly attributed to the experience RLO gained during in the training phase. The number of steps required by BO to convergence can be greatly reduced with an informed GP model, e.g. by including historic samples and data from simulations. In Fig. 3, 100 samples are used to *warm-start* the initial GP model. The samples are taken from another task, where the incoming beam was varied from the task where the evaluation is run. As expected, the performance of BO improves when historic samples are taken from a task configuration that is similar to the new task, with similarity characterised by the correlation coefficient *r*. At the extreme, where $r = {1}$, i.e. historic samples stem from the same task, convergence can even be achieved in one step. The required amount of data points for warm-starting BO prior to optimisation is four orders of magnitude lower than the samples required by RLO training, due to the sample efficiency of GP models. This makes BO an especially appealing candidate in absence of fast-executing simulations. In addition, task specific modifications can be implemented with BO to further improve the convergence speed and final tuning results. For example, if the dynamics of the tuning task are sufficiently observable, which is not the case in this study, it is possible to train an informed NN or directly use the fast-executing simulation model as a physics-informed prior mean^44–46^.

### Real-world study

In order to evaluate the methods’ ability to transfer to the real world and to verify the results obtained in simulation, we also studied the performance of RLO and BO on the ARES particle accelerator. This part of the study is crucial, as even with accurate simulations, the gap between simulation and the real world is often wide enough that algorithms performing well in simulation cannot be transferred to the real world^47^. We observed this gap between simulation and experiment in the early stages of training the RL policy, where trained policies performed well in simulation but failed altogether in the real world. Similarly, when implementing BO for the tuning task, implementations tuned for exploitation showed faster and better optimisation in simulation but failed during the experiments under real-world conditions.

**Figure 4:**
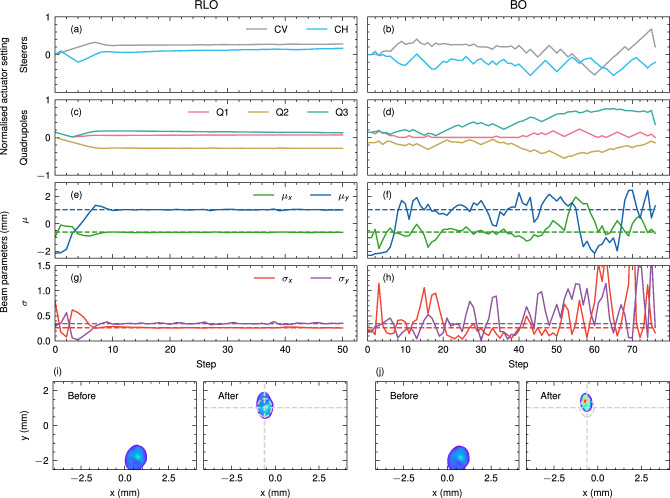
Example optimisations on the real particle accelerator. (**a**,**c**,**e**,**g**) show one optimisation with RLO. (**b**,**d**,**f**,**h**) show one optimisation with BO. (**a**,**b**) show the steering magnet settings and (**c**,**d**) show the quadrupole magnet settings. (**e**,**f**) show the beam positions and (**g**,**h**) show the beam sizes. (**i**,**j**) show the beam images before and after the optimisation respectively. The target beam size and position are indicated with dashed lines.

Given the limited availability of the real accelerator, we considered 22 trials of the 300 used for the simulation study. The magnet misalignments and the incoming beam on the real accelerator can neither be influenced nor measured during the experiment, so they were considered unknown variables. To ensure a fair comparison in benchmarking real-world performances, the algorithms are run back-to-back for each trial. As a result, the conditions experienced by different algorithms are as close as possible. Before every measurement shift, an automated alignment was performed in order to bring the incoming beam close to the centres of the quadrupole magnets and reduce dipole moments induced when the beam passes through a quadrupole magnet off-centre, which can steer the beam too far off the screen in the initial state. This adjustment is needed for BO to find an objective signal in a reasonable time. In “Robustness in the presence of sensor blind spots”, we investigate how the alignment, or the lack thereof, affects the results of this study. The results of the real-world measurements are listed in Table 1 and further illustrated in Supplementary Sect. 1. Two example optimisations by RLO and BO on the real accelerator are shown in Fig. 4. On the real particle accelerator, just like in the simulation study, we observe that RLO achieves both a better tuning result and faster convergence than BO. Both methods were able to produce final beams that were visually close to the target beam parameters, as can be seen in the beam images in (i,g). In addition, it can be observed that RLO converges more smoothly than BO (a-h). While this has little effect in simulation, in the real world, smooth convergence has various advantages which are discussed further in “Sim2real transfer”. We also compared the performance of RLO to manual tuning by expert human operators (see Supplementary Fig. S5) and found out that the trained RLO could consistently reach comparable final beam parameters faster than human operators.

In the real-world study, RLO outperforms BO in 13 out of 22 trials, which is a lower proportion compared to the simulation study. We also observe that the difference in performance between the two is not as pronounced in the real world, with the final MAE of RLO about half that of BO in the real world study, when they are an order of magnitude apart in the simulation study. While all three performance metrics of BO are almost identical between the real world and simulation, the performance of RLO appears to degrade. This is partly due to the measurement accuracy now limiting the achievable beam difference, with the result of RLO being only slightly larger at 24.46 $\upmu$m. It is important to note that to use the available machine study time most effectively, both algorithms were allowed fewer samples on the real accelerator than in simulation, and that BO was allowed more samples than RLO.

### Practical challenges

#### Sim2real transfer

The transfer of a method that works well in a simulation environment to the real world is a large part of developing tuning algorithms for facilities such as particle accelerators. The challenges posed by this so-called *sim2real* transfer impact the choice of tuning algorithm.

Successfully transferring a policy trained for RLO to the real ARES accelerator involved a number of engineering decisions^19^ detailed in “Reinforcement learning”. While some of the design choices, such as inferring changes to the actuator settings instead of the actuator settings directly, can be applied to other tuning tasks with relative ease, others, such as domain randomisation^48,49^, require specialised engineering for each considered tuning task. Furthermore, all of these require time-consuming fine-tuning to actually achieve a successful zero-shot sim2real transfer of a policy trained only in simulation. This is illustrated by the fact that many of the policies trained before the one studied here performed well in simulation while sometimes not working at all on the real ARES accelerator.

On the other hand, our BO implementation transfers to the real world with relatively little effort. Once it was sorted out how to best deal with faulty measurements, further discussed in “Robustness in the presence of sensor blind spots”, most iterations of the BO implementation performed about as well on the real accelerator as they did in simulation. Only some more specialised design decisions that appear sensible in simulation, such as tuning the acquisition function strongly towards exploitation, do not transfer well, because BO may overfit to falsely positive noisy samples of the objective function. These potential pitfalls need to be considered when first designing a BO solution in simulation. The easier sim2real transfer of BO is likely owing to the fact that GP model is learned entirely on the real observations and therefore will not overfit to a different objective function that deviates from the one under optimisation. The sim2real issue is similarly present for BO if it is warm-started using simulation data, which is less correlated to the real system. In that case, BO will initially perform better but eventually saturate at non-optimal settings. This can be mitigated by either discarding old samples or by sampling more samples from the new task. In the real world, it is therefore not always possible to warm-start BO with sufficiently correlated historic data due to drifts of the system over time.

One issue that may arise when transferring BO from simulation to the real-world task is the over-exploration behaviour. RLO naturally converges toward an optimum and then stays there, meaning that if the optimisation is ended at any time the environment’s state is at or close enough to the best-seen position as predicted by the policy. Depending on the choice of acquisition function, BO is likely to explore further even after finding the global optimum if the parameter space is large enough to encourage exploration. In practice, therefore, it is often necessary to return to the best-seen input when the optimisation is terminated. While this strategy will recover the same objective value in simulation, it is often not ideal for real-world objective functions, which are noisy, not stationary for instance due to slow thermal drifts, or path-dependent. One example is the magnet hysteresis, where the ferromagnetic core of an electromagnet retains some magnetisation when the current in its coils, on particle accelerators may shift the objective value when returning to a previously seen point in the optimisation space. In the benchmark tuning task, we experienced noisy measurements and magnet hysteresis. We found that for the studied BO trials, the final beam error deviated by a median of 11 $\upmu$m and a maximum of 42 $\upmu$m. This means that the deviation is usually smaller than the screen’s estimated accuracy. At least for the benchmark task, the effect is therefore non-negligible, but also not detrimental to the performance of the tuning algorithms. Although not considered in the scope of this study, it has been demonstrated that the hysteresis effect can be explicitly modelled in the context of BO^50^.

The path-dependent effects like magnet hysteresis are further amplified by more back-and-forth actions such as those performed by BO in the exploration phase. As already shown in Fig. 4, RLO performed much smoother actions in general, which could reduce wear on the actuators and avoid possible additional noise and shifts in the objective function. This further stabilises the tuning process and improves reproducibility. Additionally, we find that in the case of the accelerator hardware used in our particular benchmark task, erratic actions sometimes lead to glitches with the electronics of the power supplies driving the magnets. Similar challenges are encountered in imperfect real-world systems and should be avoided if possible in a deployed setting. This can be done, for example, by enforcing certain smoothness of the taken actions. One possible way for the BO case is to use the proximal biasing, which has been shown to produce smooth actions respecting the hardware limits of the accelerator^51,52^. We refer to Supplementary Sect. 3 for further details.

#### Robustness in the presence of sensor blind spots

In any real system, it is possible to encounter states where the available diagnostics deliver false or inaccurate readings, resulting in erroneous objective values and observations. Transitions to these states can be caused by external factors as well as the tuning algorithm itself. A good tuning algorithm should therefore be able to recover from these states. In the benchmark tuning task, an erroneous measurement occurs when the electron beam is not visible on the diagnostic screen within the camera’s field of view. In this case, the beam parameters computed from the diagnostic screen image are false, also resulting in a faulty objective value.

We find that when the beam is not properly observed, RLO can usually recover it in just a few steps. Presumably, the policy can leverage its experience from training to make an educated guess on the beam’s position based on the magnet settings even though faulty beam measurements were not part of its training, where RLO always had access to the correct beam parameters.

In contrast, BO struggles to recover the beam when it is off-screen initially, as the GP model is learned completely at application time. The faulty observations lead to wrong predictions of the objective and acquisition functions. Introducing constraints^51,53^ can partly mitigate this issue by marking the region with erroneous signal as *invalid* and taking steps only within the predicted safety regions, but they require either an existing model or learning the constraints online. Our implementation, as described in “Bayesian optimisation”, addresses this issue by introducing a constant punishment to the objective function when no beam is detected in the camera’s field of view. Nevertheless, the lack of information about the objective function’s topology results in BO taking many arbitrary steps before the beam is again detected on the screen and the optimisation starts progressing towards the target beam. While more comprehensive diagnostics can help solve this problem, these are often not available. Although not considered in this study, the sensor blindness issue can be alternatively mitigated by initialising the GP model with a prior mean function learned from simulation or history data, which favours the settings which result in an on-screen beam. However, this limits the transferability of BO to new problem settings, analogous to a RLO with a pre-trained policy. In addition, this is not feasible for every tuning task. For example, the considered transverse tuning task is only partially observable due to the unknown incoming beam and magnet misalignments. This makes creating an informed prior difficult and in some cases even impossible.

The presented measurements on the real accelerator were taken with the beam aligned to the quadrupole magnets in order to provide BO with an initial system state containing an informative objective signal. As a result, the additional dipole moments induced by the quadrupole magnets when increasing the magnitude of their focusing strength are kept minimal, reducing the chance that the beam leaves the camera’s field of view during the initial step of the optimisation. As this alignment would not be performed during nominal operation but may change the observed performance of both algorithms, a study was performed in simulation in order to understand how to interpret the reported results given that the beam was aligned to the centres of the quadrupole magnets before the optimisation. Both algorithms are evaluated over the same 300 trials as in “Simulation study”. Unlike in the original simulation study, we also simulate erroneous beam parameter measurements when the beam position is detected outside the camera’s field of view. Both algorithms are tested once with the original incoming beam and once with an incoming beam that was previously aligned to the quadrupole magnets. The results are reported in Table 1. We conclude that the reported results on the real particle accelerator would be expected to worsen by about 5–33% for RLO and by 12–121% for BO if the electron beam had not been aligned to the centres of the quadrupole magnets at the beginning of measurement shifts. This does not change the relative performance of the two algorithms.

#### Edge cases and limitations

Naturally, both tuning algorithms have limitations that could cause them to fail. With the goal of autonomous accelerator operation, it is important to understand these limitations and how they can be mitigated.

In the previously presented experiments, neither RLO nor BO ever produced a final beam that was worse than the beam before the optimisation. Nevertheless, as can be seen in Table 1 and Supplementary Fig. S1, the final achieved MAE varies more than an order of magnitude for both algorithms. Furthermore, there are a few outlier trials, in which the algorithms perform significantly worse. Note that these are not the same trials for both algorithms and that no clear cause of the worse performance could be identified. We assume that these outliers are likely caused by the stochastic components of both algorithms.

In order to better understand under which conditions either algorithm performs worse, we collected grid scans over the target beam space in simulation. Their results can be seen in Supplementary Fig. S8 to S12. The grid scans confirm the presence of stochastic outliers with bad performance. They further show that both learning-based algorithms perform worse for large target beams, though this effect is subtle compared to the outliers. Random search does not show the same effect. We therefore postulate that the initialisation of the magnets in a focus-defocus-focus (FDF) pattern, which is commonly used to achieve small beams, causes this bias. Note that the FDF initialisation is a conscious design choice that was made because it improved the performance of both algorithms overall.

An additional limitation can be the algorithms’ reliance on correct accelerator calibration. For example, the investigated RLO policy is trained to set the strength of the quadrupole magnet $k$ and the angle of the steering magnets. This setup is natural to accelerator physics and straightforward to integrate with beam dynamics simulations. In the real world, the accelerator control system maps quadrupole strengths and steering angles to the correct current set points for the magnets’ power supplies. This requires calibration of the beam energy. If that calibration is far off the correct beam energy, RLO may degrade in performance or even fail. For BO, accelerator calibration is mostly a non-issue, because in the case of a zero-mean prior as used in this study, BO makes no prior assumptions on the relationships of actuators to the target function. A false calibration to the beam energy would therefore be transparent to BO. In the case of beam energy calibration on magnets, this calibration is usually known well enough ahead of time for recurring working points, or the beam energy needs to be measured for other reasons anyway. However, in some other cases, where there is a need for regular recalibration, this might influence the selection of a learning-based tuning algorithm.

## Discussion

Comparing both learning-based algorithms, we find that RLO with a pre-trained policy achieves a better final tuning result and converges in fewer samples than BO. The fact that the policy is pre-trained using a computationally cheap simulation at design time makes RLO more robust to false sensor readings by leveraging experience from its training where BO learns its surrogate model from data gathered at application time, learning these faults as part of its model which then inhibits the resulting tuning. While not relevant for the specific tuning task examined in this study, RLO is three orders of magnitude faster than BO in terms of the time needed to infer the next tuning sample, making it ideal for tuning tasks where real-time decisions are required. This is due to the fact that only one forward pass of the policy is needed with RLO, where BO needs to fully optimise the acquisition function. See Supplementary Sect. 2 for more details. In contrast to the variant of BO used in this study, RLO uses its prior experience to move more directly toward the optimum, resulting in smoother operation of the actuators and avoiding issues like hysteresis and power supply failures that may result from erratic driving of real-world accelerator hardware.

These advantages of RLO, however, come at the cost of significant up-front development effort and limited transferability of the developed solution to different tuning tasks, modified versions of the same tuning task, or in some cases even different operating conditions. Training an RLO policy requires demanding engineering of reward functions and agent-environment interaction, which is often difficult and requires expertise and experience with RL. Moreover, training can take a long time and requires a lot of hyperparameter tuning to achieve optimal performance. The resulting policy is often specialised on one specific task and not guaranteed to transfer well, even if only the working point is changed or the lattice is slightly modified. To some extent, these issues can be addressed through *domain randomisation*. In fact, the RLO solution assessed in this study generalises well to different working points and drift states thanks to domain randomisation being used. We also found it to be robust to small lattice changes. Research into lattice-agnostic RL^54^ promises to address the transferability issues of RLO further.

BO on the other hand can easily be deployed to new tuning tasks thanks to the algorithm learning at the time of application. Already available and well-designed open-source implementations of BO make deploying it to accelerator tuning tasks easy and quick. In contrast to RLO, only little BO expertise is required at design time to achieve reasonably good results. More so, a working BO solution will continue to work if the working point or underlying lattice are changed, and can even be easily and quickly adapted to novel tuning tasks with decent results. With BO, as considered in this study, requiring no sample-intensive training at design time, it also forgoes the need for a high-speed simulation, making it a reliable algorithm when such a simulation is not available.

We want to note here that, while we believe the variants considered in this study are most representative of the inherent merits and challenges of either algorithm and reflect the most common ways they are implemented in particle accelerator tuning, many modifications can be made to both algorithms which may change their behaviours, strengths, and weaknesses. In some cases, these modifications can improve BO in terms that our study found to be strengths of RLO and vice versa. As mentioned before, prior information can be incorporated in the GP model by means of data-driven models or fast executing simulation models^44–46^. Similar to RLO , this can improve convergence speeds, final tuning results and robustness to faulty sensor readings at the cost of increasing the upfront engineering effort and limiting the transferability. Similarly, GP-MPC^55^ as a model-based RL approach can greatly reduce the required training time by utilising a GP to model the system dynamics. It inevitably sacrifices its fast inference time and should only be applied when the real-time requirements of the tuning task are relaxed enough. This allows direct RLO training on the real accelerator, removing the need for a high-speed simulation, but potentially makes RLO more susceptible to real-world disturbances during the training phase.

We expect comparisons of these more specialised variants of RLO and BO to be made in future work. Another interesting direction for future work is investigating both methods’ abilities to be used as feedbacks to improve the stability of particle accelerator working points. While this is historically the domain of RL, context- and time-aware BO^54,56–58^ solutions can likely handle some feedback tasks as well and may be easier to deploy, either because of the smaller design-time effort required for BO or because BO solutions might already be deployed for static tuning on the same or similar tasks.

To conclude, RLO is a strong alternative online tuning algorithm to BO, which is highly performant and clearly outperforms non-machine-learning-based approaches for particle accelerator tuning. With up to an order of magnitude better tuning results and five times faster convergence in our study, RLO deserves increased consideration, when the overall performance in terms of final tuning results, convergence speeds, inference times, and robustness to faulty sensor readings are most important, while the experiment setup is expected to remain stable and a large upfront engineering effort can be afforded. Nevertheless, BO is best used where there is little to no previous experience available, such as when optimising a completely new accelerator or accelerator setup, or when substantial distribution shifts in the experiment setup are expected, such as through lattice modifications or working point changes. BO is also suited for situations where the tuning task is only expected to be performed a few times or a tuning solution is required quickly. We believe that further research in this direction will lead to new algorithms merging the benefits of both RLO and BO approaches, allowing it to leverage the different aspects of a tuning task tailored to the actual requirements.

## Methods

To collect the data presented in this study, evaluation runs of RLO and BO as well as the baseline methods of random search, Nelder–Mead Simplex and ES were run in simulation and on a real particle accelerator. The following sections introduce the real-world plant used for our study, our experimental setups, and the optimisation algorithms.

### ARES particle accelerator section

The ARES (Accelerator Research Experiment at SINBAD) particle accelerator^39,40^, located at Deutsches Elektronen-Synchrotron DESY in Hamburg, Germany, is an S-band radio frequency linac that features a photoinjector and two independently driven travelling wave accelerating structures. These structures can operate at energies up to 154 MeV. The primary research focus of ARES is to produce and study sub-femtosecond electron bunches at relativistic energies. The ability to generate such short bunches is of great interest for applications such as radiation generation by free electron lasers. ARES is also used for accelerator component research and development as well as medical applications.

**Figure 5:**
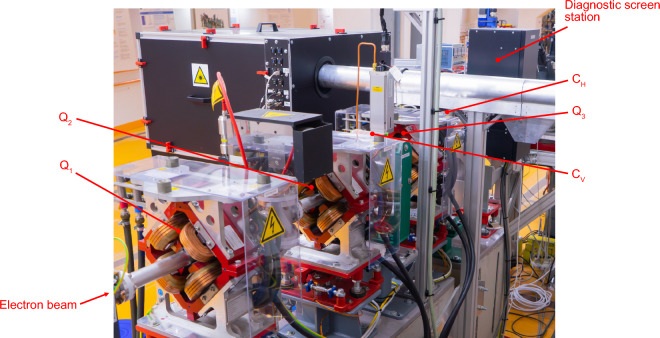
Considered accelerator section at ARES. The electron beam travels downstream from left to right. The five magnets actuated by the optimisation algorithms and the diagnostic screen station are marked.

The accelerator section, known as the *Experimental Area*, is a subsection of ARES, shown in Fig. 5 and made up of two quadrupole magnets, followed by a vertical steering magnet that is followed by another quadrupole magnet and a horizontal steering magnet. Downstream of the five magnets, there is a scintillating diagnostic screen observed by a camera. The power supplies of all magnets can be switched in polarity. The quadrupole magnets can be actuated up to a field strength of 72 m$^{-2}$. The limit of the steering magnets is 6.2 mrad. The camera observes an area of about 8 mm by 5 mm at a resolution of 2448 by 2040 pixels. The effective resolution of the scintillating screen is ca. 20 $\upmu$m.

At the downstream end of the section, there is an experimental chamber. This section is regularly used to tune the beam to the specifications required in the experimental chamber or further downstream in the ARES accelerator.

### Simulation evaluation setup

The simulation part of this study was conducted based on the *Cheetah* Python package for simulating linear beam dynamics^46,59^. Cheetah was not only used to train the RLO policies that successfully transferred to the real-world machine, but has also been evaluated in terms of its agreement with other state-of-the-art beam dynamics simulation codes through its software tests^60^.

In the simulation, a fixed set of 300 randomly generated trials were used to compare the different optimisation algorithms. Each trial is a tuple

3 $$\begin{aligned} \left( \varvec{b}', M, I \right) \end{aligned}$$

of the target beam $\varvec{b}'$ that we wish to observe on the diagnostic screen, the misalignments of the quadrupole magnets and the diagnostic screen *M*, as well as the incoming beam *I* entering the accelerator section. The target beam was generated in a range of ± 2mm for $\mu _x$ and $\mu _y$, and 0mm to 2mm for $\sigma _x$ and $\sigma _y$. These ranges were chosen to cover a wide range of measurable target beam parameters, which are constrained by the dimensions of the diagnostic screen. The incoming beam $I$ is randomly generated to represent samples from the actual operating range of the real-world accelerator. Both incoming beam and misalignment ranges were chosen to be larger than their estimated ranges present in the real machine.

### Real-world evaluation setup

In the real world, the overall machine state was set to an arbitrary normal machine state, usually by leaving it as it was left from previous experiments. This should give a good spread over reasonable working points and drift states. The target beams were taken from the trial set used for the simulation study. As the incoming beam and misalignments cannot be influenced in the real world in the same way they can be in simulation, they are left as they are on the real accelerator and considered unknown. Experiments on the real accelerator were conducted on 9 different days over the course of 82 days, running at charges between 2.6 pC and 29.9 pC, and an energy of 154 MeV. To ensure a fair comparison of the tuning methods, we align the beam to the quadrupole magnets at the beginning of each measurement day. This ensures that the beam remains within the camera’s field of view on the diagnostic screen in the initial step, which is also a common operating condition of the accelerator. Experiments for each trial were always run back-to-back, ensuring that the environmental conditions found by each algorithm were as similar as possible.

**Figure 6:**
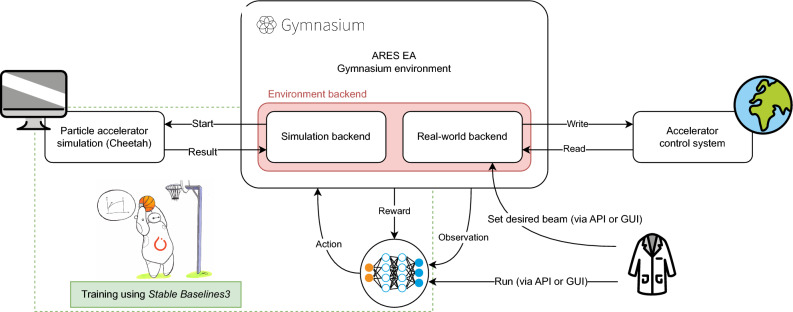
Gymnasium environment setup used for the study. In particular showing how one environment interface facilitates design and training using a simulation of the plant, as well as transferring a developed tuning algorithm to the real-world task without modification.

Transferability of the experiments between simulation and the real world as well as RLO and black-box optimisation was achieved through a combination of *OpenAI Gym*^61^ environments, an overview of which is shown in Fig. 6. These Gym environments were upgraded to *Gymnasium*^62^ environments for some later experiments. Throughout this paper, we use the terms *Gym environment* and *Gymnasium environment* interchangeably. We designed a single environment with interchangeable *backends*. The purpose of the environment is only to define the logic of the tuning task itself, i.e. the reward, the actions, and the observations. We define two different backends defining the physics of the beam tuning task. Both backends can be controlled by the environment through the same interface. One of these backends wraps around the Cheetah simulation code^46,59^, allowing for fast training and evaluation. The other backend interfaces with the accelerator’s control system. Providing the same interface for both backends ensures that the transfer from simulation to the accelerator is transparent to the agents. While Gymnasium environments are primarily designed for RL policies to interact with their task, the ones used for this work were made configurable in such a way that they can also be interfaced with a BO optimisation. This includes configurable reward formulations and action types that pass actuator settings to the step method and have the latter return an objective value via the reward field of the step method’s return tuple.

### Reinforcement learning

The RLO implementation and trained policy used for this study have been introduced in previous work^19^. In this case, a multilayer perceptron (MLP) with two hidden layers of 64 nodes each is used as a policy, observing as input the currently measured beam parameters on the diagnostic screen $\varvec{b}= \left( \mu _x, \sigma _x, \mu _y, \sigma _y \right)$, the currently set field strengths and deflection angles of the magnets $\varvec{u}= \left( k_{Q_1}, k_{Q_2}, \alpha _{C_v}, k_{Q_3}, \alpha _{C_h} \right)$, and the desired beam parameters $\varvec{b}' = \left( \mu _x, \sigma _x, \mu _y, \sigma _y \right)$ set by the human operator. The policy then outputs changes to the magnet settings $\varvec{a}_t = \Delta \varvec{u}$. A normalisation of rewards and observations using a running mean and standard deviation is performed over the training. The outputs are normalised to 0.1 times the magnet ranges of ±30 m${}^-2$ for the quadrupole magnets, ±3 mrad for the vertical steering magnet, and ±6 mrad for the horizontal steering magnet. The vertical steering magnet’s range was set to half that of the horizontal steering magnet, because it is about twice the distance from the diagnostic screen, doubling its effect on the beam position. During training and application, episodes are started from a fixed FDF setting of the quadrupole triplet, with the strengths $(k_{Q_1}, k_{Q_2}, k_{Q_3})=(10,-10,10)$ m$^{-2}$ and both steering magnets are set to 0 mrad. The policy is trained for 6,000,000 steps on the very fast-to-compute Cheetah simulation code^46,59^ using the Twin Delayed DDPG (TD3)^63^ algorithm as implemented by the *Stable Baselines3*^64^ package. TD3 was chosen for its relative training sample efficiency compared to other RL algorithms and found to produce well-performing policies. Other state-of-the-art RL algorithms such as Proximal Policy Optimisation (PPO) can however also be used to train competitive policies on the same task. Training is run in a simulation provided by the *Cheetah*^46,59^ particle tracking code, as limited availability makes training on the real particle accelerator infeasible. Domain randomisation^48^ is performed during training. Specifically, the magnet and screen misalignments, the incoming beam and the target beam are randomly sampled from a uniform distribution for each episode. The reward function used for training is

4 $$\begin{aligned} R\left( \varvec{s}_t, \varvec{a}_t\right) = {\left\{ \begin{array}{ll} \hat{R} \left( \varvec{s}_t, \varvec{a}_t \right) &{} \text {if } \hat{R} \left( \varvec{s}_t, \varvec{a}_t\right) > 0 \\ 2 \cdot \hat{R} \left( \varvec{s}_t, \varvec{a}_t\right) &{} \text {otherwise}\, \end{array}\right. } \end{aligned}$$

with $\hat{R} \left( \varvec{s}_t, \varvec{a}_t\right) = O\left( \varvec{u}_t\right) - O\left( \varvec{u}_{t+1}\right)$ and $O\left( \varvec{u}_t\right)$ being the natural logarithm of the weighted MAE between observed and target beam on the diagnostic screen. The trained policy is deployed zero-shot, i.e. without any further training or fine-tuning, to the real accelerator.

### Bayesian optimisation

The BO version used for this study was implemented using the *BoTorch*^65^ package. The goal is to maximise the negative logarithm of the weighted MAE

5 $$\begin{aligned} \max _{\varvec{u}} -O(\varvec{u}) = \max \left( -\log \left( \text {MAE}(\varvec{b}, \varvec{b}') \right) + w_\text {on-screen}\right) . \end{aligned}$$

The logarithm is used to properly weigh the fine improvement when BO approaches the target beam. A further on-screen reward $w_\text {on-screen} = 10$ is added to the objective when the beam can be observed on the screen, and subtracted from the objective to penalise the settings when the beam is off the diagnostic screen. Note that this can alternatively be achieved by incorporating a safety constraint. To increase the numerical stability of the GP regression, the previous input settings $\varvec{u}$ are min-max normalised to $[-1,1]$ and objective values are standardised. The covariance function of the GP models used in this study is the sum of a Matérn-5/2 kernel^66^ and a white noise function. The GP hyperparameters, like the length scales and signal noise, are determined dynamically by log-likelihood fits in each step. In each trial, BO is started from the same fixed FDF setting used by RLO. Five random samples are taken to initialise the GP model. Based on the posterior prediction of the GP model, an Expected Improvement (EI)^67^ acquisition function is calculated, which automatically balances the exploration and exploitation of the objective. The next sample is chosen by maximising the acquisition function. The maximum allowed step sizes are constrained to 0.1 times the total action space, which is the same setting that is used for RLO. In practice, to avoid the time-consuming polarity changes of the quadrupole magnets’ power supplies, the quadrupole magnets are only allowed to vary in the same polarity, i.e. in the FDF setting. This does not hamper the final performance in simulation studies, as the target beam parameters can still be achieved in this reduced action space. BO is allowed to run 150 steps in simulation and 75 steps on the real machine, after which we return to the best settings found. We have tested other BO hyperparameter settings, for example, using Upper Confidence Bound (UCB) as an acquisition function, and they lead to a comparable performance. Note that our model choices were mainly decided using the simulation before deploying it to the real accelerator, reducing the required beam time for the model development.

The variant of BO used in this study is shown to match the performance of other state-of-the-art implementations such as those provided by the popular *Xopt* package^68^. For comparisons of the performance of our implementation to those provided by Xopt, please refer to “Discussion”.

### Random search

For the random search baseline, we sample random magnet settings from the constrained space of magnet settings. Constraint in this case means that we limit the space to a range commonly used during operations, instead of the full physical limits of the magnets. The latter limits are almost an order of magnitude larger than anything ever used in operation. At the end of the optimisation, we return to the best example found.

### Nelder–Mead simplex

The Nelder–Mead Simplex optimisation^42^ was implemented using the *SciPy*^69^ Python package. The initial simplex was tuned in a random search of 405 samples to the one that performed best across the set of 300 trials. Nelder–Mead is allowed to run for a maximum of 150 steps. After 150 steps or after early termination the simplex algorithm might perform better if it returns to the final sample, but as it is generally converging, it does not necessarily need to. The objective function optimised by Nelder–Mead is the MAE of the measured beam parameters to the target beam parameters.

### Extremum seeking

We used the implementation of the ES algorithm provided by the *Xopt* Python package, based on the description in^70^. The update of the input parameter $p_i$ in step *n* follows

6 $$\begin{aligned} p_i (n+1) = p_i (n) + \Delta _t \sqrt{\alpha _i \omega _i} \cos (\omega _i n \Delta _t + k_i C(n), \end{aligned}$$

where *C* is the objective value and $\alpha _i$ the amplitude.

According to the original paper, we normalised the input parameters to $[-1,1]$, selected the dithering amplitudes $\omega _i = r_i \omega _0$ with $r_i$ linearly spanned in the range [1.0, 1.75], and chose $\Delta _t = \frac{2\pi }{10 \max {\omega _i}}$. Extensive hyperparameter tuning was performed using Bayesian optimisation across the full set of trials to minimise the best MAE after 150 steps. The evaluated ES algorithm uses the following settings: feedback gain $k={3.7}$, oscillation size $s={0.11}$ (scales the oscillation amplitude in Eq. (6)), and decay rate $\lambda ={0.987}$, where the default settings are $\{k={2.0}, s={0.1}, \lambda ={1.0}\}$.

Furthermore, in our optimisation study, we used an exponential decay on the oscillation amplitude to improve the convergence to the target beam parameters. While the original implementation used ES without decay to achieve stable long-term control, the decay rate helps convergence when using ES in an optimisation setting. Tuning the oscillation amplitude without the usage of a decay rate $\lambda$ leads either to local optimisation or large oscillation at the final steps, resulting in a worse final MAE.

**Table 1:** Performance of optimisation algorithms on the benchmark tuning task.

Optimiser	Final beam difference ($\upmu$m)		Steps to target			Steps to convergence	
	Median	Mean	Median	Mean	Success rate	Median	Mean
Simulation (infinite diagnostic screen, no beam alignment) $\rightarrow$ Section “Simulation study”							
Random search	460	${490} \pm {200}$	–	–	0%	28	${48} \pm {50}$
Nelder–Mead simplex	270	${300} \pm {160}$	56	${55} \pm {7}$	1%	28	${25} \pm {14}$
Extremum seeking	81	${111} \pm {94}$	121	${107} \pm {38}$	17%	43	${47} \pm {29}$
RLO	4	${11} \pm {19}$	7	${12} \pm {16}$	92%	6	${7} \pm {10}$
BO	45	${60} \pm {55}$	40	${48} \pm {26}$	43%	19	${29} \pm {26}$
Real world (finite diagnostic screen, beam aligned to quadrupole magnets) $\rightarrow$ Section “Real-world study”							
RLO$^{a,b}$	24	${29} \pm {48}$	12	${13} \pm {7}$	55%	7	${8} \pm {4}$
BO$^{a,c}$	44	${58} \pm {41}$	45	${48} \pm {12}$	45%	13	${17} \pm {11}$
Simulation (finite diagnostic screen, no beam alignment) $\rightarrow$ Section “Robustness in the presence of sensor blind spots”							
RLO	4	${13} \pm {40}$	7	${12} \pm {16}$	93%	6	${7} \pm {4}$
BO	38	${61} \pm {96}$	44	${53} \pm {28}$	53%	20	${30} \pm {30}$
Simulation (finite diagnostic screen, beam aligned to quadrupole magnets) $\rightarrow$ Section “Robustness in the presence of sensor blind spots”							
RLO	4	${10} \pm {18}$	6	${12} \pm {15}$	95%	6	${6} \pm {3}$
BO	23	${28} \pm {19}$	33	${37} \pm {19}$	85%	19	${20} \pm {11}$

The results reported in simulation have been collected over 300 different trials, for each of which the algorithms were given 150 steps for the optimisation. Because of limited beam time availability at the real accelerator, 22 trials (a) were selected for the real-world evaluations, RLO was given 50 steps(b) for the optimisation and BO was given 75 steps (c) for the optimisation. The final beam difference is computed as the MAE over the beam parameters at the end of the optimisation according to Eq. (2). Steps to target are computed as the number of steps it took until an MAE to the target beam smaller than the threshold $\epsilon =$ 40 $\upmu$m was achieved. Steps to convergence are defined as the number of steps after which the improvement of the best seen MAE remains smaller than the threshold $\epsilon$. For each metric, we report the median and the mean with standard deviation. The steps to target and steps to convergence metrics are reported only over trials for which the target was achieved before the optimisation was terminated. We therefore also report the success rate, that is the proportion of trials for which the target was successfully achieved before termination.

### Supplementary Information

Supplementary Information.

## Data Availability

The data generated for the presented study is available at https://doi.org/10.5281/zenodo.7853721.

## Code availability

The code used to conduct and evaluate the presented study is available at https://github.com/desy-ml/rl-vs-bo.
