# Supplementary material for: Reinforcement learning-trained optimisers and Bayesian optimisation for online particle accelerator tuning
Source: Sci Rep. 2024 Jul 8;14:15733. doi: 10.1038/s41598-024-66263-y (PMC11231256; doi:10.1038/s41598-024-66263-y)
Supplement: Supplementary file 1 — Supplementary Information. [file 41598_2024_66263_MOESM1_ESM.pdf]

Supplementary Information

Reinforcement Learning-trained Optimisers and Bayesian Optimisation for Online Particle Accelerator Tuning

Jan Kaiser[1,*], Chenran Xu[2,+], Annika Eichler[1,3], Andrea Santamaria Garcia[2], Oliver Stein[1], Erik Bründermann[2], Willi Kuropka[1], Hannes Dinter[1], Frank Mayet[1], Thomas Vinatier[1], Florian Burkart[1], and Holger Schlarb[1]

[1]Deutsches Elektronen-Synchrotron DESY, Germany
[2]Karlsruhe Institute of Technology KIT, Germany
[3]Hamburg University of Technology, 21073 Hamburg, Germany
[*]jan.kaiser@desy.de
[+]chenran.xu@kit.edu

1 Performance metrics box plots

The following box plots give a better sense of the distributions of the final beam errors, steps to target and steps to convergence for each algorithm.

Figure 1 shows the MAE at the end of each optimisation by the different algorithms. It can be seen that just like Bayesian optimisation (BO), reinforcement learning-trained optimisation (RLO) outperforms the non-learning algorithms. RLO also outperforms BO, both in simulation and in the real world. We can observe that this performance difference is still large, but not quite as pronounced in the real world. The latter effect is further discussed in Sec. Real-world study.

In Fig. 2, RLO is the only algorithm to reliably get within the target threshold of $\varepsilon = 40\,\mu\text{m}$ before the maximum number of steps, though a significant proportion of optimisation runs performed by BO also achieve the target in time. The non-learning algorithms mostly don't achieve the target in the maximum number of steps, with extremum seeking (ES) achieving an mean absolute error (MAE) within the threshold in a small proportion of trials, Nelder-Mead Simplex achieving the target 3 times, and random search never getting there.

Figure 3 shows box plots of the number of steps taken until the optimisations by each algorithm converged. This does not necessarily mean that they converged on the target, but rather that the MAE did not change by more than $\varepsilon = 40\,\mu\text{m}$. We observe that all algorithms almost always converge within the allowed number of steps. With respect to Fig. 2, this suggests that they converge to a local optimum rather than the global one. As would be expected, this effect is especially pronounced with Nelder-Mead simplex optimisation, which converges almost a quickly as RLO and BO, but almost never gets within the threshold around the target. The effect is much more subtle with RLO and BO, suggesting that they often find the global optimum, or at least a local optimum that is close to the global optimum in its objective value.

2 Inference times

The time it takes to infer the next set of actuator settings may also influence the algorithm choice. For the benchmark task, the inference time happens to be negligible, because our benchmarked physical system, specifically the magnets and the beam measurement, is orders of magnitude slower than the inference time. At other facilities, where the physical process takes less time, the time taken for tuning may be dominated by the inference time of the tuning algorithm and there might even be real-time requirements[1].

Inference times for RLO and BO can vary greatly depending on the choice of model and other design parameters. Nevertheless, we performed inference time measurements on the specific implementations of RLO and BO used in our study. We measure the average inference times of both algorithms over the 45 000 inferences of the simulation study using a MacBook Pro with an M1 Pro chip running Python 3.9.15. It is observed that BO takes an average of 0.7 s to infer the next actuator settings, while RLO is more than three orders of magnitude faster at 0.0002 s. This reflects the generally expected trend that RLO is capable of faster inference because the RLO policy requires only one forward pass of the multilayer perceptron (MLP) with a complexity of $O(1)$ with respect to the steps taken. By contrast, in each BO inference step, a full optimisation of the acquisition function is performed. This involves inferences with the GP model with complexity $O(n^3)$, scaling with the number of steps taken n. Even when choosing a different faster model, BO requires an optimisation of the acquisition function in each step, meaning it is generally expected to have slower inference than RLO. Note that the RLO inference can also be sped up significantly by using specialised hardware[2].

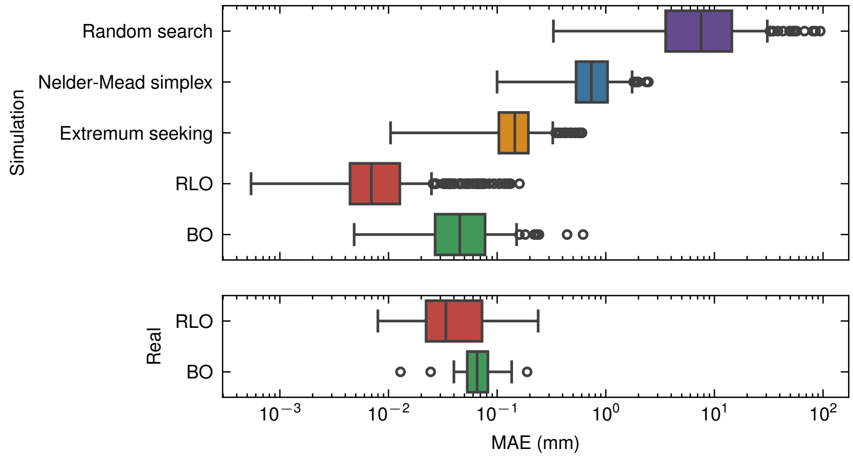

Supplementary Figure S1. Final beam distances achieved by different algorithms. Given as the MAE to the target beam when the optimisation was terminated. The boxes show the interquartile range with a vertical line indicating the value of the median. The whiskers indicate the rest of the final beam distances as 1.5 times the interquartile range and outliers outside that range are indicated by black markers.

3 Bayesian optimisation implementation performance

With the number of different variants and implementations of BO that are available, it is not trivial to choose which to use for evaluations such as those presented in the main paper. We chose to use a state-of-the-art BO implementation described in Sec. Bayesian optimisation of the main paper. To ensure that our implementation matches the state-of-the-art that has evolved for the particle accelerator community, we compare our version to the Xopt package. For benchmarking purposes, we evaluated two BO versions from the Xopt backend, one uses a hard step-size constraint and the other one uses proximal-biasing[3] as a soft step-size constraint. Both use upper confidence bound (UCB) acquisition with $\beta = 2$ and perform the default pre-processing steps, i.e. normalising the input to $[0,1]$ and standardising the objective values. The hard version used the same step-size limit of 0.1 as the BO and RLO versions used in this study. The proximal weight was set to be 0.5, which means the acquisition drops to $\frac{1}{e}$ over 10 % of the action space. This was obtained from a hyperparameter tuning, optimising for the best MAE within 150 steps. Note that this is higher than the original value 0.1, which was used for a much smoother objective landscape. In the studied ARES task, smaller proximal weights would mostly lead to premature convergence, due to a large number of local optima as shown in Fig. 6 and Fig. 7.

Figure 4 shows the results of our comparison. While in simulation, we find that our implementation falls right in between the two provided by Xopt, on the real ARES accelerator, our implementation consistently performs the best. We therefore conclude that our implementation is representative of the state of the art in BO for particle accelerator tuning. Nevertheless, it needs to be mentioned that the proximal BO produced smoother action than the other versions, which could be favourable for other tasks where smooth actions are more critical.

4 Comparison to expert human operators

The three policies trained with different random seeds for the RLO implementation have been compared to two expert human operators on a single trial, with tuning runs being conducted consecutively. Note that RLO can only interact with the accelerator every 10 s to 20 s because it has to wait for magnets to reach their set points and a new beam measurement to be taken before predicting the next action. The human operators are not limited by this and can already take the next action while the magnets are settling and a trend can be identified, thus being able to interact with the accelerator at more than 1 Hz. The results indicate that while achieving a similar final beam as the expert human operators, RLO can do so faster and more consistently, despite having a much slower interaction rate. A plot of the MAE developing over the tuning by RLO compared to the human operators is shown in Fig. 5.

5 Optimisation space example

In the following two figures, we show parts of the objective space for one instance of the considered particle accelerator tuning task. This helps develop an intuition for the shape of the objective function and the way it is explored by RLO and BO. Note that the two figures show different slices of the objective function, as they are shown with respect to the final sample

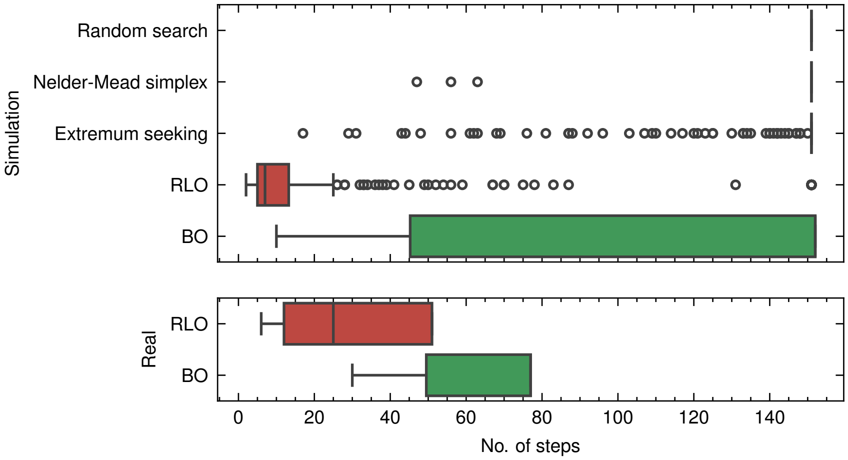

Supplementary Figure S2. Number of steps taken by different algorithms to either reach the target within $\varepsilon = 40\,\mu\text{m}$ or be terminated. The boxes show the interquartile range with a vertical line indicating the value of the median. The whiskers indicate the rest of the final beam distances as 1.5 times the interquartile range and outliers outside that range are indicated by black markers. For *Random search*, *Nelder-Mead simplex*, and *Extremum seeking*, the boxes are drawn only as thin lines because almost all optimisation runs are terminated at their respective step limit.

found by each algorithm. It can be seen that both algorithms successfully find the optimum without exploring large regions of the objective space. Further, the fewer samples taken by RLO also cover a much smaller region of the objective space than those taken by BO. They draw a relatively direct line to the optimum found by RLO, supporting the assumption that RLO can make use of experience from training to know in which direction to find the optimum. As is suggested by the slightly different performance achieved by RLO and BO, the algorithms converged on slightly different locations in the objective space. Encouragingly, the optima found by both are relatively close to each other.

6 Grid scans over target beams

In an effort to better understand the relationships between the environment's state and optimiser performance, grid scans were performed over the position and size of the target beam. Their results are visualised in Figs. 8 to 12. The target beams were scanned with 20 samples for each of the 4 beam parameters, resulting in 160 000 different target beams. Misalignments and incoming beams were kept constant for these scans. As expected, the random search showed comparable results for all the trials regardless of the target beam parameters. Whereas for both RLO and BO, small target beams generally resulted in slightly better MAEs. To verify that this is not the result of the random seed used when training the particular policy considered for the study presented in the main manuscript, two additional policies were trained with the same setup but different random seeds. They exhibit the same behaviour, as shown in Figs. 9 and 10. The cause of this effect is discussed in Sec. Edge cases and limitations.

References

1. Wang, W. *et al.* Accelerated deep reinforcement learning for fast feedback of beam dynamics at KARA. *IEEE Transactions on Nucl. Sci.* **68**, 1794–1800, DOI: 10.1109/TNS.2021.3084515 (2021).

2. Scomparin, L. *et al.* KINGFISHER: A Framework for Fast Machine Learning Inference for Autonomous Accelerator Systems. In *Proc. 11th Int. Beam Instrum. Conf. (IBIC'22)*, no. 11 in International Beam Instrumentation Conference, 151–155, DOI: 10.18429/JACoW-IBIC2022-MOP42 (JACoW Publishing, Geneva, Switzerland, 2022).

3. Roussel, R. *et al.* Turn-key constrained parameter space exploration for particle accelerators using bayesian active learning. *Nat. Commun.* **12**, 5612 (2021).

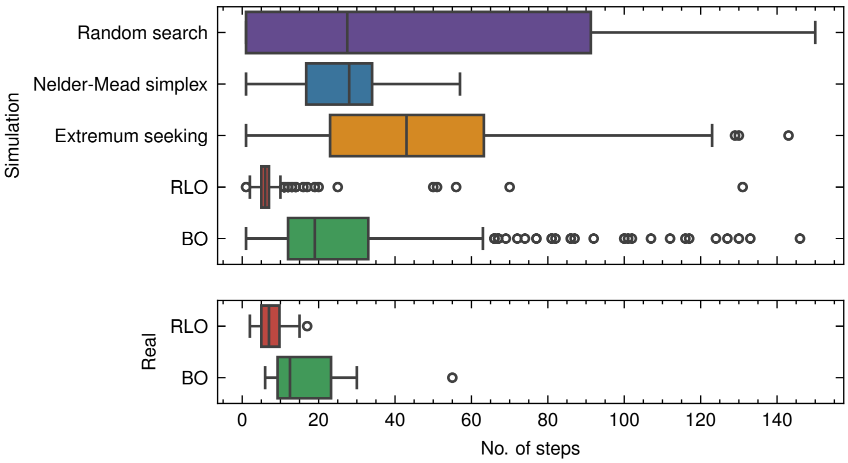

Supplementary Figure S3. Number of steps taken by different algorithms until convergence or termination. Convergence is defined as the time steps from which on the maximum future change is smaller than $\varepsilon = 40\,\mu\text{m}$. The boxes show the interquartile range with a vertical line indicating the value of the median. The whiskers indicate the rest of the final beam distances as 1.5 times the interquartile range and outliers outside that range are indicated by black markers.

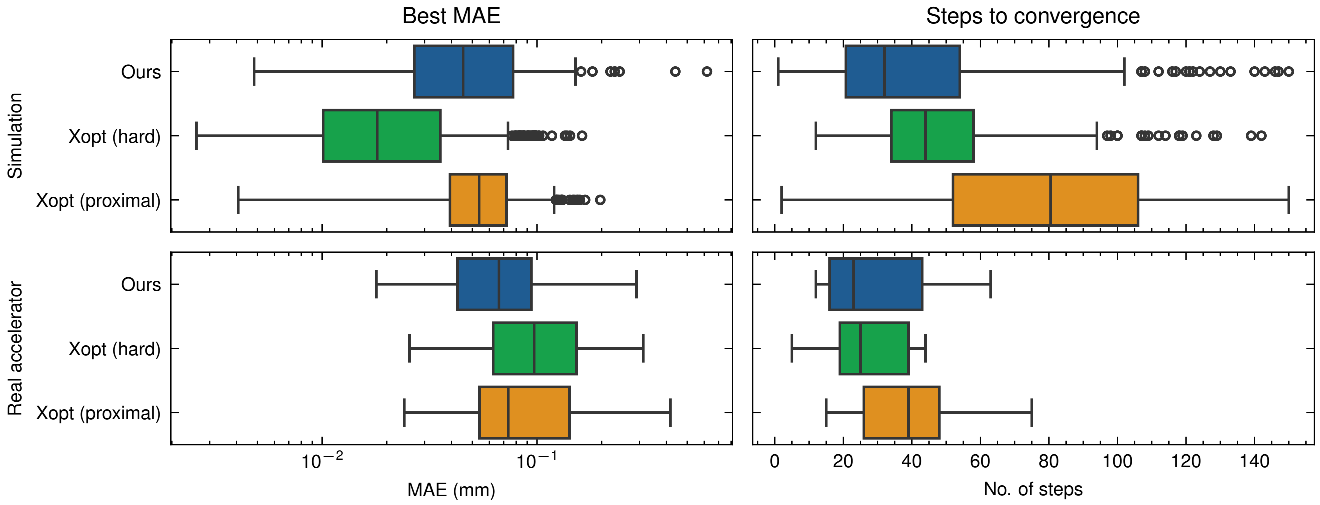

Supplementary Figure S4. Comparison of the BO implementation used in our evaluations to two other state-of-the-art implementations provided by *Xopt*. The two plots on the left show the best MAE achieved during the optimisation procedure, while the plots on the right show the number of steps it took for the best seen MAE to converge. The top row shows results obtained from evaluations in simulation. Results obtained on the real ARES accelerator are shown in the bottom row.

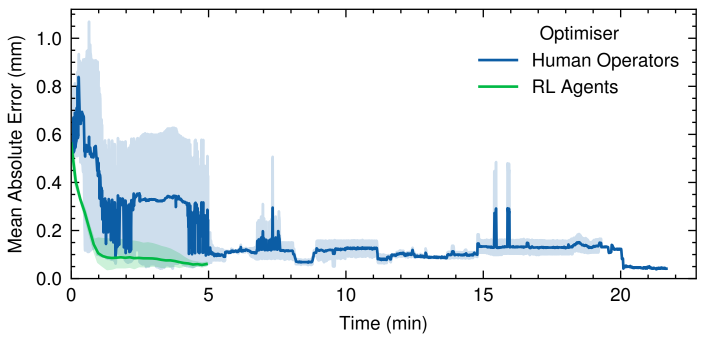

Supplementary Figure S5. Comparison of RLO to expert human operators. Showing distance from the observed beam to the target beam over time. The mean beam distance as the MAE is shown by the solid line. The envelope shows the 95 % confidence interval of the beam distance.

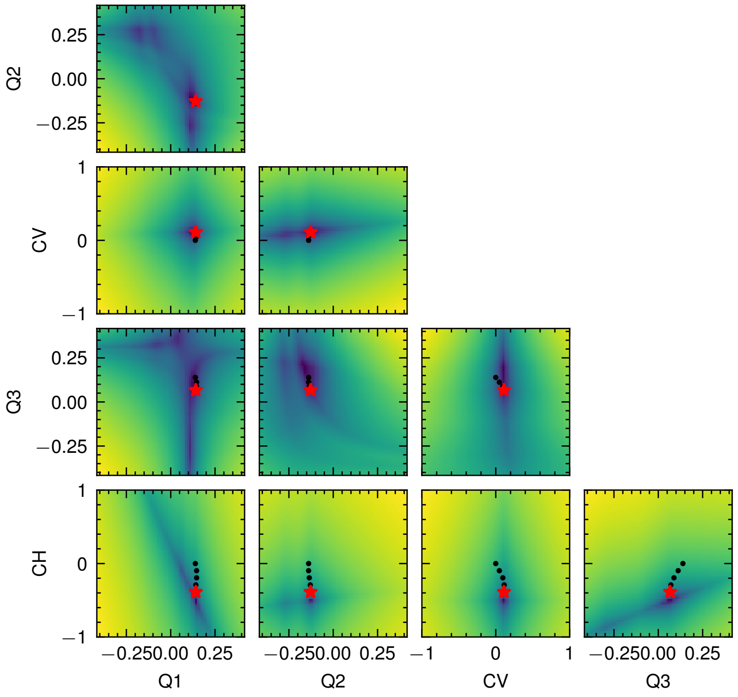

Supplementary Figure S6. Samples taken by RLO on the objective function. The trial shown here is the same as in Manuscript Fig. 3, but in simulation. Black dots mark samples taken by the algorithm before the threshold ε was reached. Each heat map shows a two-dimensional slice of the objective space anchored in the final sample, denoted by the red star. All magnet settings are normalised to their permissible ranges, with the final normalised magnet setting at $\left(k_{Q_1}, k_{Q_2}, \alpha_{C_v}, k_{Q_3}, \alpha_{C_h}\right) = (0.14, -0.13, 0.11, 0.067, -0.39)$. The colour maps are scaled individually for each slice, and shown logarithmically for easier illustration. The colour yellow indicates a large objective value. Dark blue indicates a small objective value.

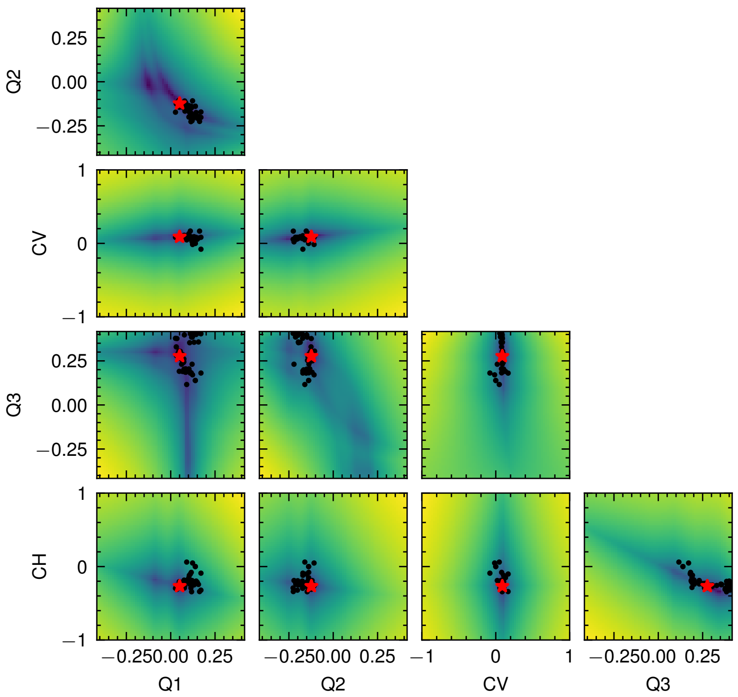

Supplementary Figure S7. Samples taken by BO on the objective function. The trial shown here is the same as in Manuscript Fig. 3, but in simulation. Black dots mark samples taken by the algorithm before the threshold ε was reached. Each heat map shows a two-dimensional slice of the objective space anchored in the final sample, denoted by the red star. All magnet settings are normalised to their permissible ranges, with the final normalised magnet setting at $\left(k_{Q_1}, k_{Q_2}, \alpha_{C_v}, k_{Q_3}, \alpha_{C_h}\right) = (0.050, -0.12, 0.088, 0.28, -0.27)$. The colour maps are scaled individually for each slice, and shown logarithmically for easier illustration. The colour yellow indicates a large objective value. Dark blue indicates a small objective value.

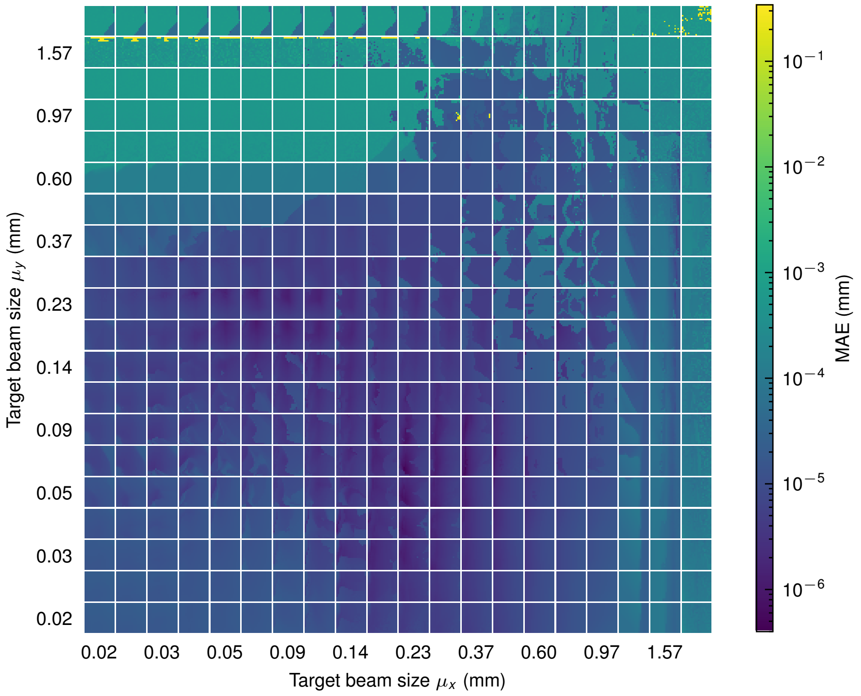

Supplementary Figure S8. Grid scan over target beams using the RLO policy evaluated in the main manuscript. The scan was performed over 160 000 different target beams with fixed incoming beam and misalignments. Beam positions in x and y are visualised within each box, with the diagnostic screen's centre in their centre and the beam position increasing linearly outwards. Beam sizes are visualised across boxes, starting with beam size 20 µm in the bottom left and increasing logarithmically to the top right. The colour map is scaled logarithmically.

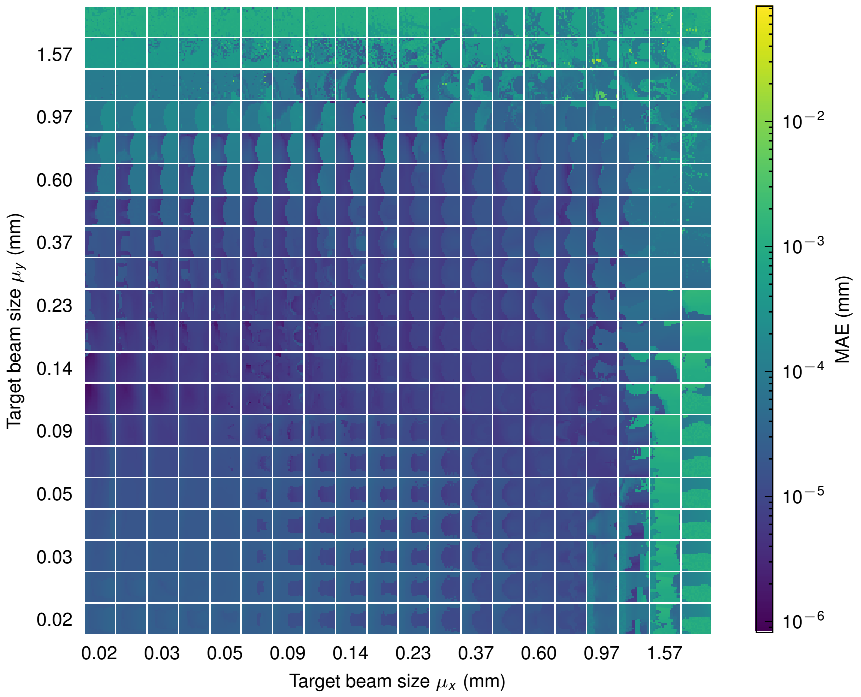

Supplementary Figure S9. Grid scan over target beams using an RLO policy trained with a different random seed than the one evaluated in the main manuscript. The scan was performed over 160 000 different target beams with fixed incoming beam and misalignments. Beam positions in x and y are visualised within each box, with the diagnostic screen's centre in their centre and the beam position increasing linearly outwards. Beam sizes are visualised across boxes, starting with beam size 20 µm in the bottom left and increasing logarithmically to the top right. The colour map is scaled logarithmically.

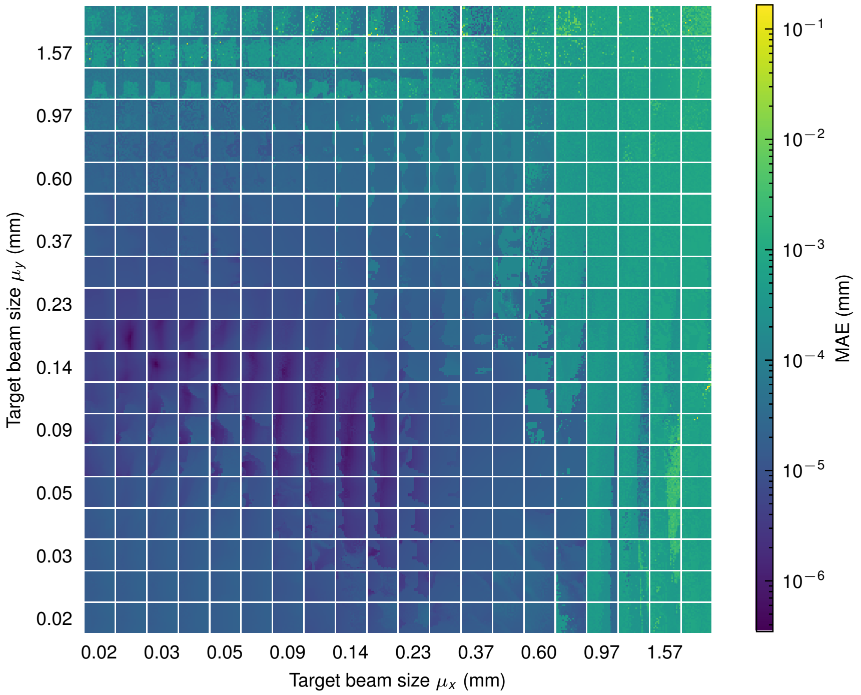

Supplementary Figure S10. Grid scan over target beams using a second RLO policy trained with a different random seed than the one evaluated in the main manuscript. The scan was performed over 160 000 different target beams with fixed incoming beam and misalignments. Beam positions in x and y are visualised within each box, with the diagnostic screen's centre in their centre and the beam position increasing linearly outwards. Beam sizes are visualised across boxes, starting with beam size 20 µm in the bottom left and increasing logarithmically to the top right. The colour map is scaled logarithmically.

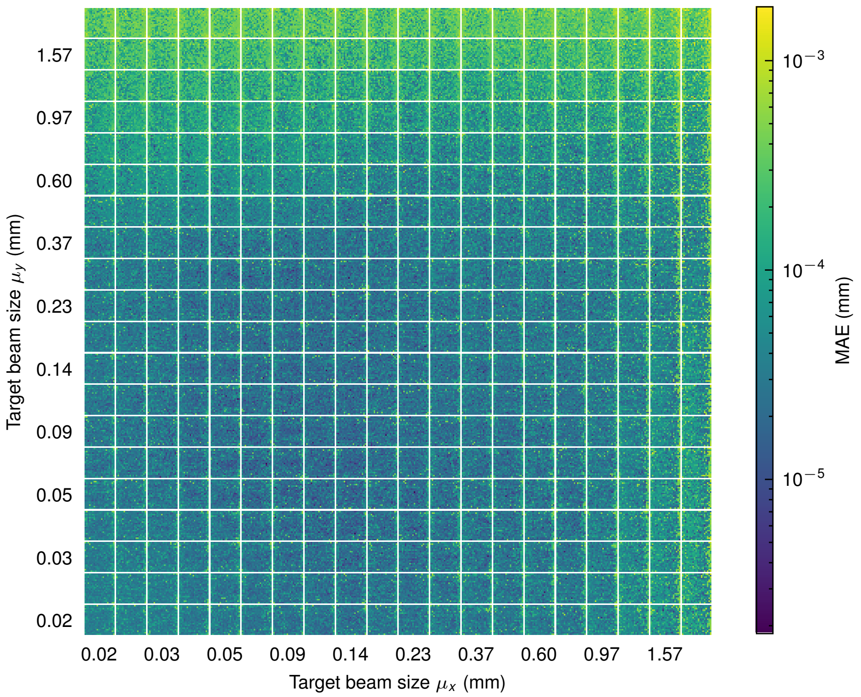

Supplementary Figure S11. Grid scan over target beams using BO. The scan was performed over 160 000 different target beams with fixed incoming beam and misalignments. Beam positions in x and y are visualised within each box, with the diagnostic screen's centre in their centre and the beam position increasing linearly outwards. Beam sizes are visualised across boxes, starting with beam size 20 µm in the bottom left and increasing logarithmically to the top right. The colour map is scaled logarithmically.

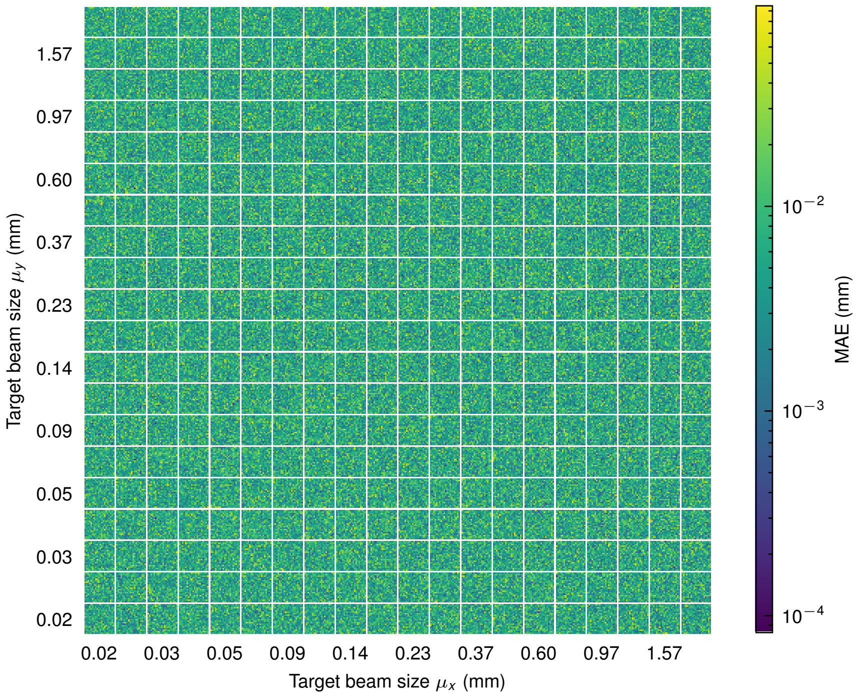

Supplementary Figure S12. Grid scan over target beams using random search. The scan was performed over 160 000 different target beams with fixed incoming beam and misalignments. Beam positions in x and y are visualised within each box, with the diagnostic screen's centre in their centre and the beam position increasing linearly outwards. Beam sizes are visualised across boxes, starting with beam size 20 µm in the bottom left and increasing logarithmically to the top right. The colour map is scaled logarithmically.
